# Unilateral spatial neglect in the acute phase of ischemic stroke can predict long-term disability and functional capacity

**DOI:** 10.6061/clinics/2018/e131

**Published:** 2018-05-15

**Authors:** Gustavo José Luvizutto, Augusta Fabiana Moliga, Gabriela Rizzo Soares Rizzatti, Marcelo Ortolani Fogaroli, Eduardo de Moura, Hélio Rubens de Carvalho Nunes, Luiz Antônio de Lima Resende, Rodrigo Bazan

**Affiliations:** I Departamento de Fisioterapia Aplicada, Universidade Federal do Triangulo Mineiro (UFTM), Uberaba, MG, BR; II Departamento de Reabilitacao, Faculdade de Medicina de Botucatu (UNESP), Botucatu, SP, BR; III Graduacao, Faculdade de Medicina de Botucatu, Botucatu, SP, BR; IV Departamento de Fisioterapia, Faculdade de Talentos Humanos (FACTHUS), Uberaba, MG, BR; V Departamento de Estatistica, Faculdade de Medicina de Botucatu (UNESP), Botucatu, SP, BR; VI Departamento de Neurologia, Psicologia e Psiquiatria, Faculdade de Medicina de Botucatu (UNESP), Botucatu, SP, BR

**Keywords:** Stroke, Unilateral Spatial Neglect, Functional Independence, Incapacity, Autonomy

## Abstract

**OBJECTIVE:**

The aim of this study was to assess the relationship between the degree of unilateral spatial neglect during the acute phase of stroke and long-term functional independence.

**METHODS:**

This was a prospective study of right ischemic stroke patients in which the independent variable was the degree of spatial neglect and the outcome that was measured was functional independence. The potential confounding factors included sex, age, stroke severity, topography of the lesion, risk factors, glycemia and the treatment received. Unilateral spatial neglect was measured using the line cancellation test, the star cancellation test and the line bisection test within 48 hours of the onset of symptoms. Functional independence was measured using the modified Rankin and Barthel scales at 90 days after discharge. The relationship between unilateral spatial neglect and functional independence was analyzed using multiple logistic regression that was corrected for confounding factors.

**RESULTS:**

We studied 60 patients with a median age of 68 (34–89) years, 52% of whom were male and 74% of whom were Caucasian. The risk for moderate to severe disability increased with increasing star cancellation test scores (OR=1.14 [1.03–1.26], *p*=0.01) corrected for the stroke severity, which was a confounding factor that had a statistically positive association with disability (OR=1.63 [1.13–2.65], *p*=0.01). The best chance of functional independence decreased with increasing star cancellation test scores (OR=0.86 [0.78–0.96], *p*=0.006) corrected for the stroke severity, which was a confounding factor that had a statistically negative association with independence (OR=0.66 [0.48–0.92], *p*=0.017).

**CONCLUSION:**

The severity of unilateral spatial neglect in acute stroke worsens the degree of long-term disability and functional independence.

## INTRODUCTION

Stroke is both the second leading cause of death worldwide and the primary cause of chronic disability in adults 1–5. Unilateral spatial neglect (USN) is characterized by the inability to respond to people or objects that are presented contralateral to the lesioned side of the brain and is also a symptom that cannot be accounted for by either motor or sensory deficits. USN is more common in right- than left-hemisphere strokes and can contribute to disability 6,7. The incidence of USN varies widely, ranging from 10–82% in right-hemisphere stroke patients 8-10. The main areas involved in USN are related to the right hemisphere, such as lesions in the right posterior parietal lobe 11-12, and individuals with USN after stroke present with major functional disabilities as well as decreased rates of adherence to rehabilitation programs 13-15. Furthermore, USN can decrease a patient’s ability to return to work and thus has socioeconomic impacts on a community’s public health status 16,17. The aim of this study was to evaluate the relationship between the degree of USN during the acute phase of stroke with disability and long-term functional independence. The main hypothesis of this study was that a higher degree of USN during the acute phase of a stroke with disability would predict greater long-term disability.

## MATERIALS AND METHODS

### Study design and participants

This observational study was conducted in accordance with the principles of the Declaration of Helsinki. Patients were selected after the study protocol was approved by the institutional review board of the Botucatu Medical School (Of. 122/11). All participants or their legal representatives were aware of the study objectives and provided written informed consent.

This study included 60 individuals who had suffered a right-hemisphere stroke, as confirmed by a CT or MRI scan, with a cut-off Mini-Mental State Examination score of >24. The individuals were admitted to the stroke unit of the Botucatu Medical School between March 2015 and April 2016. We excluded patients with previous cranial trauma, hemorrhagic stroke, dementia, prior changes in vision, hemianopsia or other associated neurological diseases.

### Measurement of unilateral spatial neglect and long-term outcomes

The degree of USN was measured during the acute phase of stroke, which was between 48 and 72 hours after the stroke onset, using three tests.

1) The line cancelation test (LCT): the degree of USN was determined by the proportion of lines that were omitted from a total of 40 lines randomly distributed on one sheet of paper 18. A greater omission of lines indicated more severe USN.

2) The star cancelation test (SCT): the degree of neglect was determined by the proportion of stars that were omitted from a total of 56 stars that were associated with distractors 19. A greater omission of stars indicated more severe USN.

3) The line bisection test (LBT): each patient was asked to detect and indicate the point corresponding to the midlines of 18 transverse lines that were arranged in three columns (at the left, center, and right of the page) of six lines each. The degree of neglect was determined by the location of the selected point relative to the midline 20. A greater deviation of the selected point from the midline indicated more severe USN.

In all the USN tests, the examiner placed the examination sheet in front of the patient with a distance of 60 cm between the glabella and the center of the paper 21.

The following are the potential confounding factors that could have affected the outcome of this study: age, sex, race, years of education, risk factors, topography (LACS=lacunar stroke; PACS=partial anterior circulation; TACS=total anterior circulation; POCS=posterior circulation), etiology (large-vessel occlusion, small-vessel occlusion, cardioembolism, other causes, or indeterminate), National Institutes of Health Stroke Scale (NIHSS) score at admission, blood glucose level at admission and treatment received. The risk factors evaluated included hypertension, smoking, obesity, diabetes mellitus (DM), alcohol consumption, dyslipidemia, prior stroke, congestive heart failure (CHF), prior acute myocardial infarction (AMI), atrial fibrillation and depression. Additional data, including any previous use of antihypertensive medications, oral hypoglycemic agents, parenteral insulin or oral lipid-lowering drugs, were either collected from the clinical history of each patient or were confirmed clinically by laboratory tests administered during hospitalization. Hypertension was indicated when the systolic blood pressure was ≥140/90 mmHg, dyslipidemia was indicated when cholesterol levels were ≥240 mg/dL, DM was indicated when the glycated hemoglobin level was >7%, obesity was indicated when the body mass index was ≥30 kg/m^2^, and depression was indicated by a score >8 on the Hospital Anxiety and Depression Scale (HADS) 22-25.

The long-term outcomes examined in this study were functional independence, as measured by the modified Rankin scale (mRS), and autonomy, as evaluated by the Barthel scale 26. The outpatient follow-up time was 90 days, and the outcomes were evaluated by the principal investigator of the study. Outcomes were classified as favorable if the patient presented an mRS score of 0–2 or unfavorable if the patient presented an mRS score of 3–5. The autonomy outcome was considered favorable if the Barthel index score was greater than 95 27. All evaluated patients underwent physiotherapy, which consisted of conventional exercises, with the rehabilitation service at the Botucatu Medical School.

### Sample size

Because a sample of the target population was selected from a specific source, the sample type was considered nonprobabilistic. A previous study by our research group determined that it would be necessary to evaluate 50 patients to achieve a statistical power of 80% (beta error 0.2 and alpha error 0.05) 28.

### Statistical analysis

Multiple logistic regression was used to analyze the effect of USN on disability and autonomy, and potential confounders were adjusted for by backward selection of data with a value of *p*>0.1. In the adjusted multiple regression model, statistical significance was set at *p*<0.05. Statistical analyses were performed using SPSS software version 21.0 (IBM^®^, Chicago, Illinois, USA).

## RESULTS

**Figure 1:**
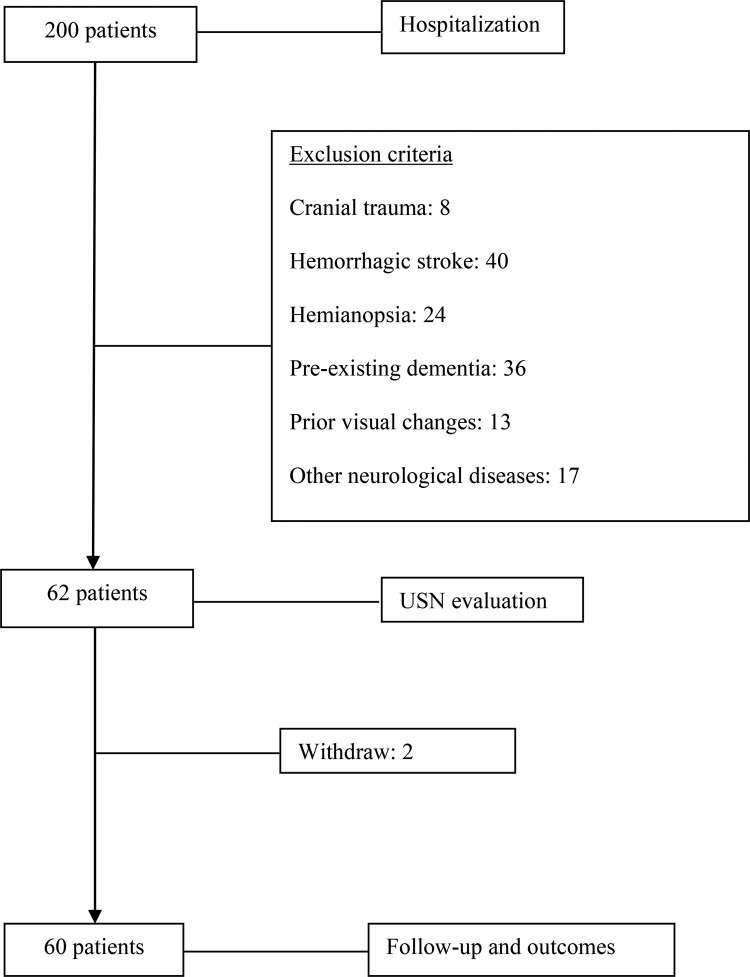
Flow diagram of the patients included in the study.

Sixty (60) of the 200 individuals recruited for the study met the eligibility criteria. The main reasons for exclusion of an individual were presentation of cranial trauma (n=8), previous hemorrhagic stroke (n=40), hemianopsia (n=24), previous presentation of dementia (n=36), prior changes in vision (n=13) and other neurological diseases (n=17). Two patients discontinued participation in the study during the follow-up period (Figure 1).

The clinical and demographic data of the patients are displayed in Table 1. The median age was 68 years, and the patients were predominantly male and white. Hypertension was the main risk factor, and the clinical-topographic classification was predominantly PACS with cardioembolic etiology. The mean blood glucose level at entry was 114 mg/dL, and the average NIHSS score was 11. Among the patients studied, 21 received conservative treatment, 9 underwent intravenous thrombolysis, and 5 underwent a decompressive hemicraniectomy.

In the univariate analyses of the mRS (Table 2) and the Barthel index (Table 3), the only variables that were correlated with the outcome were the NIHSS score and the SCT score.

The risk of moderate to severe 3–5 disability, as measured by the mRS, increased with increasing SCT scores (OR=1.14 [1.03–1.26], *p*=0.010); this result was corrected for the effect of potential confounders. The NIHSS score was a confounder with a significantly positive association with disability (OR=1.63 [1.13–2.65], *p*=0.010; Table 4).

The likelihood of functional independence, as measured by the Barthel scale, decreased with increasing SCT scores (OR=0.86 [0.78–0.96], *p*=0.006); this result was corrected for the effect of potential confounders. The NIHSS score was a confounder with a significantly negative association with independence (OR=0.66 [0.48–0.92], *p*=0.017; Table 5).

## DISCUSSION

This study demonstrated that the degree of acute-stage USN is an important predictor of long-term disability in patients who have experienced a right-sided stroke. It is well established in the literature that the NIHSS score on admission affects the functional outcome of patients with ischemic stroke. However, in the subgroup of patients with USN, several factors may interfere with the functional outcome 29,30. The degree of USN is related to the region of the ischemic or hemorrhagic lesion, and higher degrees of USN have been reported in patients with lesions in the posterior parietal region, which interfere with the attention network and, consequently, diminish the performance of functional activities 31,32.

Our study is novel in three ways. First, we adjusted our data for the NIHSS confounder, whereas authors of previous studies have not tested for this correlation 33-34. Higher NIHSS scores are associated with extensive brain damage in the acute phase and a poorer prognosis in the chronic phase of stroke. High NIHSS scores in our study were associated with poor outcomes, and this result suggests that stroke severity affects recovery. Second, we used the mRS to determine the functional prognosis. This scale is widely used to measure functional outcomes in large clinical trials of stroke patients. Third, we tested the correlation between LCT or LBT scores and the functional prognosis of individuals with a right-hemispheric stroke.

The SCT was the best predictor of long-term disability in our study. Several authors have reported that cancelation tasks are generally the most sensitive tests and that the SCT is the most reliable test for measuring the degree of USN at any stage of stroke because of its high sensitivity and specificity, whereas the LBT has relatively poor sensitivity 13,35. The SCT is the strongest predictor of disability in patients who have experienced a stroke in the right hemisphere, with an efficacy similar to that of the NIHSS; this finding is internationally recognized by the scientific community.

In this study, we did not investigate the mechanisms underlying the unfavorable prognostic role of USN in the chronic phase, such as those involved in trunk control, postural balance and stroke volume, because the role of USN in this phase could be related to highly neglected findings and could influence the observed results. However, this work emphasizes the need for physicians to consider USN as an important prognostic factor in ischemic stroke. USN is neglected by the major neurological scales; if a patient with only USN presents during the acute phase of ischemic stroke, the treatment protocols do not consider initiating thrombolytic therapy as they do for aphasic patients. The importance of USN should be considered in protocols for the evaluation and treatment of the acute phase of stroke.

In conclusion, patients presenting with more severe USN during the acute phase of a right-hemisphere ischemic stroke have a poorer prognosis in terms of functional independence and long-term autonomy than do patients with less severe USN.

## AUTHOR CONTRIBUTIONS

Luvizutto GJ was responsible for the literature search, data collection, and manuscript writing. Moliga AF and Rizzatti GR were responsible for manuscript writing and data collection. Neto EM and Fogaroli MO were responsible for the data interpretation. Nunes HR was responsible for the data analysis and interpretation. Resende LA was responsible for the data interpretation and study design. Bazan R was responsible for the study design.

## Figures and Tables

**Table 1:** Description of the sample.

Variable	N=60	%
**Demographic**		
Male	31	51.6
Age (years)^1^	68 (34–89)	
**Race**		
Caucasian	48	80.0
Non-Caucasian	12	20.0
**Years of education**	7 (1 – 10)	
**Risk factors**		
Hypertension	45	75.0
Diabetes	18	30.0
Smoking	22	36.6
Obesity	8	13.3
Alcoholic	8	13.3
Prior stroke	5	8.3
CHF	2	5.7
Prior AMI	4	3.3
AF	8	13.3
Depression	8	13.3
**BAMFORD**		
LACS	6	10.0
PACS	30	50.0
TACS	20	33.3
POCS	4	6.7
**TOAST**		
Large-artery atherosclerosis	15	25.0
Cardioembolism	21	35.0
Small-vessel occlusion	6	10.0
Other determined etiology	6	10.0
Undetermined etiology	12	20.0
**Glycemia at admission**^1^	114 (71–140)	
**NHISS at admission**^1^	11 (3–24)	
**Treatment**		
Conservative	35	58.3
Thrombolysis	16	26.6
Surgery	9	15.0

1 Values are presented as the median; CHF=congestive heart failure; AMI=acute myocardial infarction; AF=atrial fibrillation; LACS=lacunar stroke; PACS=partial anterior circulation; TACS=total anterior circulation; POCS=posterior circulation; NIHSS=National Institutes of Health Stroke Scale.

**Table 2:** Univariate analyses comparing patients with favorable *vs*. unfavorable modified Rankin scale scores.

Variables	Modified Rankin Scale cut-off		
	0-2 (n=19)	3-5 (n=41)	*p*
**Demographic**			
Median Age	66.0 (44.0 - 88.0)	68.0 (34.0 - 89.0)	0.503
Non-Caucasian	3 (15.8%)	9 (22.0%)	0.735
Median years of education	6 (1-9)	7 (1-10)	0.335
**Risk factors**			
Hypertension	12 (63.2%)	33 (80.5%)	0.103
Diabetes	6 (31.6%)	12 (29.3%)	0.856
Smoking	7 (36.8%)	15 (36.6%)	0.985
Obesity	4 (21.1%)	4 (9.8%)	0.249
Alcoholic	2 (10.5%)	6 (14.6%)	1.000
Prior stroke	1 (5.3%)	4 (9.8%)	1.000
CHF	1 (5.3%)	1 (2.4%)	0.537
Prior AMI	1 (5.3%)	3 (7.3%)	1.000
AF	4 (21.1%)	4 (9.8%)	0.249
Depression	3 (15.8%)	5 (12.2%)	0.699
Glycemia at admission	114.0 (87.0 - 140.0)	114.0 (71.0 - 140.0)	0.663
NIHSS at admission	6.0 (3.0 - 11.0)	13.0 (4.0 - 24.0)	<0.001
LCT	10.0 (0.0 - 28.0)	30.0 (6.0 - 40.0)	0.056
SCT	22.0 (6.0 - 40.0)	40.0 (16.0 - 52.0)	<0.001
LBT	42.3 (15.7 - 72.7)	78.3 (29.8 - 98.8)	0.068

CHF=congestive heart failure; AMI=acute myocardial infarction; AF=atrial fibrillation; NIHSS=National Institutes of Health Stroke Scale; LCT=line cancellation test; SCT=star cancellation test; LBT=line bisection test.

**Table 3:** Univariate analyses comparing patients with favorable *vs*. unfavorable Barthel index scores.

Variables	Barthel Cut-off		
	<95 (n=6)	≥95 (n=54)	
**Demographic**			
Median age	61.0 (44.0 - 76.0)	68.5 (34.0 - 89.0)	0.056
Non-Caucasian	0 (0%)	12 (22.2%)	0.333
Median years of education	6 (1-9)	7 (1-10)	0.399
**Risk factors**			
Hypertension	4 (66.7%)	41 (77.4%)	0.620
Diabetes	2 (33.3%)	16 (29.6%)	1.000
Smoking	1 (16.7%)	21 (38.9%)	0.400
Obesity	0 (0.0%)	8 (14.8%)	0.585
Alcoholic	1 (16.7%)	7 (13.0%)	1.000
Prior stroke	1 (16.7%)	4 (7.4%)	0.421
CHF	0 (0.0%)	2 (3.7%)	1.000
Prior AMI	0 (0.0%)	4 (7.4%)	1.000
AF	1 (16.7%)	7 (13.0%)	1.000
Depression	0 (0.0%)	8 (14.8%)	0.585
Glycemia at admission	96.0 (87.0 - 137.0)	114.0 (71.0-140.0)	0.056
NIHSS at admission	8.0 (.0 - 11.0)	11.0 (3.0 - 24.0)	<0.001
LCT	11.5 (2.0 - 28.0)	25.0 (0.0 - 40.0)	0.074
SCT	23.0 (14.0 - 40.0)	37.0 (6.0 - 52.0)	0.048

CHF=congestive heart failure; AMI=acute myocardial infarction; AF=atrial fibrillation; NIHSS=National Institutes of Health Stroke Scale; LCT=line cancellation test; SCT=star cancellation test; LBT=line bisection test.

**Table 4:** Model adjusted to explain the chance of moderate to severe disability 90 days after a stroke, as measured by the mRS as a function of the star cancellation test.

Variable	β	SE	*p*	OR	95% CI	
NIHSS	0.49	0.19	0.010	1.63	1.13	2.35
SCT	0.13	0.05	0.010	1.14	1.03	1.26
Constant	-8.41	2.89	0.004	0.00		

NIHSS=National Institutes of Health Stroke Scale; SCT=star cancellation test; β=beta estimate; SE=standard error; OR=odds ratio; CI=confidence interval.

**Table 5:** Model adjusted to explain the chance of autonomy 90 days after a stroke, as measured by the Barthel index as a function of the star cancellation test.

Variable	β	SE	*p*	OR	95% CI	
NIHSS	0.40	0.17	0.017	0.66	0.48	0.92
SCT	0.14	0.05	0.006	0.86	0.78	0.95
Constant	-7.63	2.65	0.004	0.00		

NIHSS=National Institutes of Health Stroke Scale; SCT=star cancellation test; β=beta estimate; SE=standard error; OR=odds ratio; CI=confidence interval.
